# Anxiety, a significant risk factor for coronary artery disease: what is the best index

**DOI:** 10.1186/s12888-024-05798-w

**Published:** 2024-06-14

**Authors:** Mojtaba Rezaee, Haniyeh Darroudi, Leila Etemad, Arya Nasimi Shad, Zahra Zardast, Houra Kohansal, Majid Ghayour-Mobarhan, Fatemeh Sadeghian, Mohsen Moohebati, Habibollah Esmaily, Susan Darroudi, Gordon A. Ferns

**Affiliations:** 1 Department of Psychiatry, Faculty of Medicine, Mashhad University of Medical Sciences, Mashhad, Iran; 2 Binaloud University of Mashhad, Mashhad, Iran; 3 Pharmaceutical Research Center, Pharmaceutical Technology Institute, Mashhad University of MedicalSciences, Mashhad, Iran; 4 Medical Toxicology Research Center, Faculty of Medicine. Mashhad University of Medical Sciences, Mashhad, Iran; 5 Student Research Committee, Faculty of Medicine, Mashhad University of Medical Sciences, Mashhad, Iran; 6 Student Research Committee, School of Medicine, Shahid Beheshti University of Medical Sciences, Tehran, Iran; 7 Biochemistry Department, Ghaem Hospital, Mashhad University of Medical Sciences, Mashhad, Iran; 8 Department of Nutrition, Mashhad University of Medical Sciences, Mashhad, Iran; 9 Metabolic Syndrome research center, Mashhad University of Medical Sciences, Mashhad, Iran; 10 Department of Biochemistry and Biophysics, Faculty of Sciences, Mashhad Branch, Islamic Azad University, Mashhad, Iran; 11 Department of Cardiovascular, School of Medicine, Mashhad University of Medical Sciences, Mashhad, Iran; 12 Department of Biostatistics, School of Health, Mashhad University of Medical Sciences, Mashhad, Iran; 13 Social Determinants of Health Research Center, Mashhad University of Medical Sciences, Mashhad, Iran; 14 Vascular and Endovascular Surgery Research Center, Mashhad University of medical sciences, Mashhad, Iran; 15 Brighton and Sussex Medical School, Division of Medical Education, Sussex BN1 9PH, U, Falmer, Brighton, UK

**Keywords:** Coronary artery diseases, Anxiety, Factor structure model, Beck anxiety inventory, Panic disorder

## Abstract

**Background:**

Coronary artery disease (CAD) is known as the leading cause of disability and death globally. Anxiety disorders are also recognized as common types of mental disorders that substantially impact global health. Iran ranks among the countries with a high incidence of CAD and anxiety disorders. Therefore, the present study aims to determine the potential association and epidemiological aspects of anxiety and CAD within the population of Mashhad, the second most popoulos city in Iran.

**Methods:**

The present study is based on extracted data from the Mashhad stroke and heart atherosclerotic disorder (MASHAD) study which is a 10-year prospective cohort study intended to assess the effects of various CAD risk factors among Mashhad city residents. Anxiety scores were assessed at the baseline using Beck Anxiety Inventory and individuals were classified based on the BAI 4-factor structure model which included autonomic, cognitive, panic, and neuromotor components. Accordingly, the association between baseline anxiety scores and the BAI four-factor model with the risk of CAD events was analyzed using SPSS software version 21.

**Results:**

Based on the results, 60.4% of the sample were female, and 5.6% were classified as having severe forms of anxiety. Moreover, severe anxiety was more prevalent in females. Results showed a 1.7% risk of CAD (*p*-value < 0.001) over 10 years with one unit increase in anxiety score. Based on the 4-factor model structure, we found that only panic disorder could significantly increase the risk of CAD by 1.1% over the 10-year follow-up (*p*-value < 0.001).

**Conclusion:**

Anxiety symptoms, particularly panic disorder, are independently and significantly associated with an increased overall risk of developing CAD over a 10-year period. Therefore, further studies are warranted to investigate the mechanisms through which anxiety may cause CAD, as well as possible interventions to mitigate these processes.

## Introduction

Cardiovascular diseases (CVDs), with a worldwide prevalence of 573 million, were estimated to account for about 32% of deaths in 2017 [1, 2]. Reported disability-adjusted life years (DALYs) due to CVDs have doubled since 1990; solidifying their status as the leading cause of disability and death globally [1]. Notably, Iran ranks among the countries with the highest age-standardized prevalence of CVDs, with more than 9,000 cases per 100,000 individuals [3]. Additionally, it has been shown that CVDs are responsible for 45.45% of all-cause mortality in Iran [4], signaling a significant transition in the leading causes of death among Iranian people from diarrheal and infectious diseases to CVDs in the last few decades [5]. Coronary heart disease (CHD) may subsequently cause serious complications such as arrhythmias, cardiogenic shock, pericardial effusion, pulmonary embolism, and heart failure [6].

Anxiety is defined as a temporary fear, concern, and ambiguity regarding the future, that varies among individuals on its depth and repetition. In severe forms, it can be considered as a class of anxiety disorders including panic disorder, generalized anxiety disorder, separation anxiety disorder, phobias, etc [7]. Anxiety disorders are among the common types of mental disorders with a substantial impact on global health. According to the Global Burden of Disease Study 2019, the worldwide prevalence of different types of anxiety disorders is up to 300 million; accompanied by an approximate incidence of 45 million [8]. It was reported that the lifetime prevalence of common mental health disorders is up to 29.2% [9]. The prevalence of anxiety disorders in Iran has been estimated as high as 15.6% [10]. Additionally, it has been shown that patients with anxiety are more susceptible to various comorbidities such as gastrointestinal, cardiorespiratory, endocrine, and neurologic illnesses [11].

Anxiety disorders are found to be more prevalent in coronary artery disease (CAD) patients compared to the general population [12]. Although the causal relationship between anxiety disorders and CVDs has not yet been established, a growing number of studies are showing the possible unfavorable effect of anxiety on CVDs, particularly on CAD [13], which is a major and most important subtype of CVDs. Moreover, as already mentioned, both anxiety disorder and CAD exert considerable impact on morbidity, mortality, and quality of life, reinforcing the necessity of additional investigations.

Given that the coexistence of anxiety and CAD may contribute to a higher number of disabilities and mortalities, and considering the limited knowledge of its epidemiological aspects in the Mashhad (the second-most-populous city in Iran) population, the present study aims to determine the association between anxiety and factor models of BAI items with CAD in Mashhad.

## Materials and methods

### Study population

The present study is based on extracted data from the Mashhad stroke and heart atherosclerotic disorder (MASHAD) study which is a 10-year prospective cohort study intended to assess the effects of numerous CAD risk factors such as nutrition, environment, psychological disorders, and genetics on CAD events among Mashhad city residents. Participants were recruited from three regions of Mashhad, the second most populated city of the country located in north-eastern Iran (2016 Census), using a stratified cluster random sampling method. The inclusion criteria were (I) between 35 and 65 years of age; (II) having the physical and mental capability of participating in the clinical examination; (III) having no specific plans to leave the area after the first and second stages of the study. All individuals were well-informed, and their written consent was drawn. During the 10-year follow-up, of the initial 9,704 study participants, 1715 subjects were lost during follow-up due to lack of response, reluctance, lack of access, and migration. Moreover, 429 subjects passed away, leaving 7560 individuals included in the data analysis, and 7389 subjects were recruited in this study (171 missing data in anxiety) [14].

**Fig. 1:**
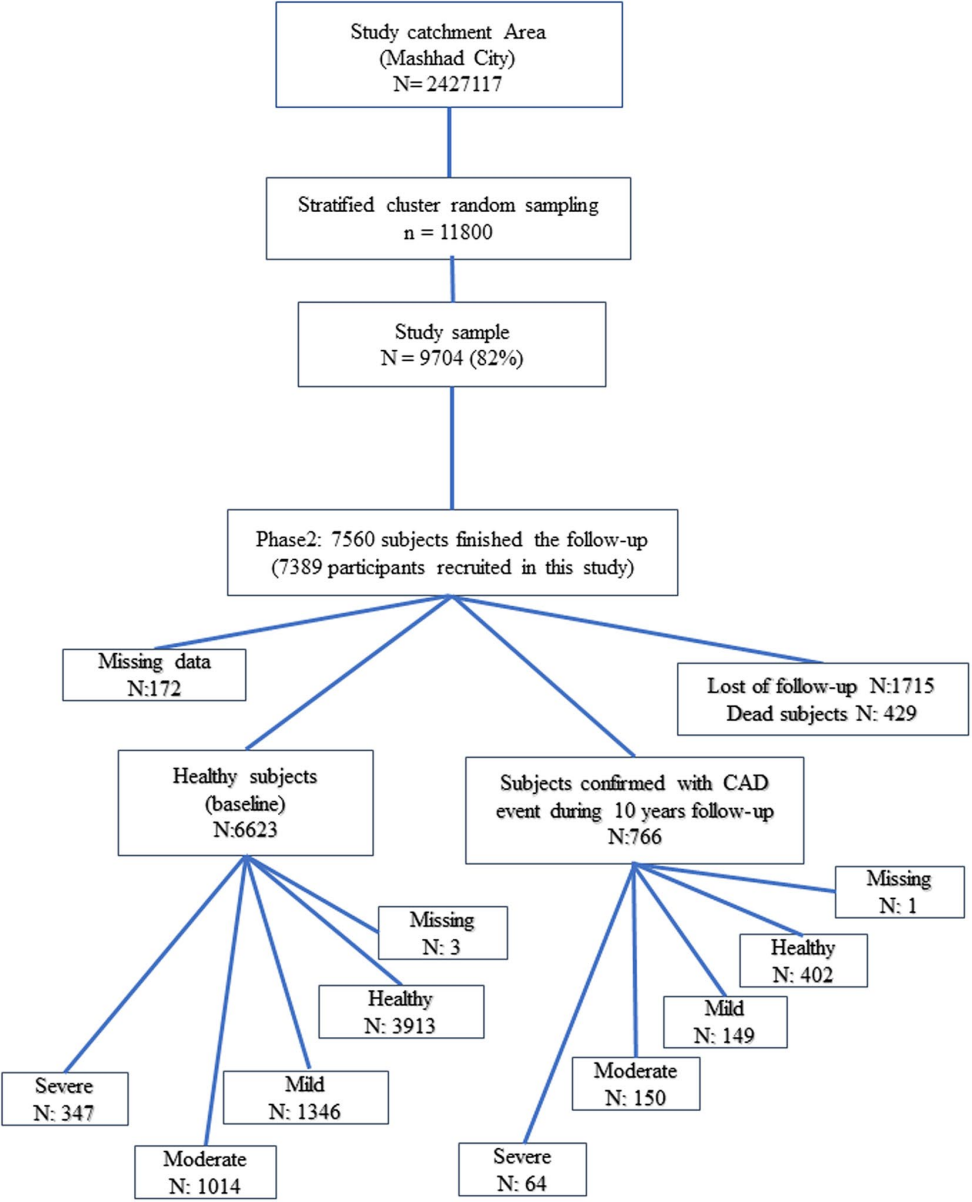
Flowchart of study subjects

Individuals were classified into 2 groups based on their cardiovascular health state including healthy and CAD. The 1st group had reported no documented coronary artery disease during the 10-year follow-up and was considered a healthy group (*N* = 6623) whilst the 2nd group had documented and confirmed CAD (*N* = 766) (Fig. 1). Healthy and CAD groups were further subdivided into 4 categories based on their psychological state and Beck Anxiety Inventory [15] which included, mild, moderate, and severe anxiety as well as healthy group (Fig. 1)(Table 1). All individuals were well informed and their written consent was drawn. Accordingly, the study protocol was validated by the Ethics Committee of the Mashhad University of Medical Sciences (MUMS) and the Institutional Review Board of Mashhad University Medical Center.

**Table 1:** Demographics and clinical features according to anxiety categorized in MASHAD study at baseline

		Anxiety				
		No (0–9)	Mild (10–16)	Moderate (17–29)	Severe (30–63)	*p*-value
N, %7389		4315 (58.4%)	1495 (20.2%)	1169 (15.8%)	411 (5.6%)	
Age		48.2±8.23	48.01±8.25	48.14±8.33	47.55±8.22	0.15
Sex	Male 2928 (39.6)	2023 (69.1%)	501 (17.1%)	311 (10.6%)	93 (3.2%)	< 0.001
	Female 4457 (60.4%)	2292(51.4%)	994 (22.3%)	853 (19.1%)	318 (7.1%)	
Marriage	Single, 40(0.5%)	19 (47.5%)	9 (22.5%)	10 (25%)	2 (5%)	0.001
	Married, 6949(94.1%)	4109 (59.1%)	1387 (20%)	1070 (15.4%)	383 (5.5%)	
	Divorced, 86 (1.2%)	37 (43%)	22 (25.6%)	20 (23.3%)	7 (8.1%)	
	Widow, 310(4.2%)	150 (48.4%)	77 (24.8%)	64 (20.6%)*	19 (6.1%)	
Smoking	No, 5217(70.6%)	3150 (60.4%)	1038 (19.9%)	762 (14.6%)	267 (5.1%)	< 0.001
	Former, 663(9%)	389 (58.7%)	128 (19.3%)	112 (17%)	33 (5%)	
	Current, 1505 (20.4%)	776 (51.6%)	329 (21.9%)	289 (19.2%)	271 (7.4%)*	
Job status	Employed, 2836 (38.4%)	1873(66%)	517 (18.2%)	337 (11.9%)	109 (3.8%)	< 0.001
	Unemployed, 3862 (52.3%)	2000 (51.8%)	839 (21.7%)	743 (19.2%)*	280 (7.3%)	
	Retired, 684 (9.3%)	440 (64.3%)	138 (20.2%)	84 (12.3%)	22 (3.2%)	
Education level	Low, 3887 (52.7%)	2195 (56.5%)	796 (20.5%)	659 (17%)	237 (6.1%)	< 0.001
	Moderate, 2640 (35.8%)	1549 (58.7%)	531 (20.1%)	414 (15.7%)	146 (5.5%)	
	High, 852 (11.5%)	569 (66.8%)*	166 (19.5%)	90 (10.6%)	27 (3.2%)	
Clinical feature						
Obesity	No, 5154 (69.9%)	3145 (61%)	1026 (19.9%)	718 (13.9%)	265 (5.1%)	< 0.001
	Yes, 2220 (30.1%)	1161 (52.3%)	467 (21%)	446 (20.1%)*	146 (6.6%)	
Diabetes	No, 6380 (87.6%)	3780 (59.2%)	1273 (20.0%)	992 (15.5%)	335 (5.3%)	0.015
	Yes, 907 (12.4%)	495 (54.6%)	195 (21.5%)	151 (16.6%)	66 (7.3%)*	
Hypertension	No, 5207 (70.7%)	3089 (59.3%)	1061 (20.4%)	790 (15.2%)	267 (5.1%)	< 0.001
	Yes, 2159 (29.3%)	1215 (56.3%)	430 (19.9%)	372 (17.2%)	142 (6.6%)*	
CAD	No, 6620 (89.6%)	3913 (59.1%)	1346 (20.3%)	1014 (15.3%)	347 (5.2%)	< 0.001
	Yes, 766 (10.4%)	402 (52.5%)	149 (19.5%)	151 (19.6%)	64 (8.4%)*	

Chi-square test or one-way ANOVA has been done; data presented as Mean ± SD or number and percentage

*CAD* coronary artery disease

### Demographic, anthropometric, and metabolic data

Sociodemographic data were collected from all participants through a questionnaire as described before [14], which included marital status, socioeconomic, job, and smoking status, and education level. Baseline anthropometric data including height, weight, waist and hip circumference, body mass index(BMI), systolic and diastolic blood pressure, and biochemical and hematological measurements were also gathered from all participants as formerly explained [14],

For all subjects, height (in cm), weight (in kg), and body mass index (in kg/m2) were measured. Height and weight were measured in centimeters and the nearest 0.1 cm with a stadiometer (SECA 217, Hamburg, Germany) and calibrated digital balance in kilogram scale (SECA 813, Hamburg, Germany) to the nearest 0.1 kg, respectively. Subsequently, BMI was calculated and categorized into obese (BMI ≥ 30 kg/m2) and non-obese (BMI < 30 kg/m2) subjects. Systolic and diastolic blood pressure (SBP and DBP) were measured using a standard sphygmomanometer, twice in the same manner. BP was measured using the left arm with individuals remaining seated after 15 min. We took the third measurement and averaged the two closest readings, if the first two readings differed by > 15mmHg for DBP or > 25mmHg for SBP. HTN was defined in accordance with the International Diabetic Federation (IDF) criteria for a SBP ≥ 130 mmHg and/or DBP ≥ 85 mmHg and/or if antihypertensive medication was used. Diabetes mellitus was defined as a fasting plasma glucose ≥ 126 mg/dL, or being under treatment with oral hypoglycemic agents or insulin. A total subjects who declared symptoms of CAD were invited to the clinic and visited by an expert cardiologist. Patients were checked and the diagnosis was confirmed by utilizing stress echocardiography, radioisotope scan, coronary angiography, computed tomography (CT), or exercise tolerance test (ETT). In ten years follow-up, 17.5% CABG, 6.7% MI, 44.4% PCI, 23.3% stable angina and 8.1% unstable angina were confirmed.

### Measurement of anxiety

The Beck Anxiety Inventory [15] was exerted for evaluating anxiety symptoms in this study [16]. It is a 21-question inquiry that investigates the frequency of the patient’s anxiety symptoms [17]. Questions are based on a 4-point Likert scale and each one may attain a score from 0 to 3. The cumulative score can range from 0 to 63 and is divided into 4 levels to determine the severity of anxiety [16]. Kaviani et al. demonstrated that the Persian-translated edition of BAI retains assuring validity (*r* = 0.72, *P* < 0.001), reliability (*r* = 0.83, *P* < 0.001), and internal consistency (Alpha = 0.92) [18]. Additionally, Clark et al. investigated various factor structure models for the BAI among CVD patients and suggested the four-factor model as a more compatible method in this population [19]. It was based on autonomic, cognitive, panic, and neuromotor components which we also applied to our classification method [19, 20].

### Statistical analyses

SPSS software version 21 was used to analyze the data (IBM SPSS, Inc., Armonk, NY, USA). The Kolmogorov-Smirnov test was used to determine the normality of the data. Descriptive analysis, qualitative and quantitative variables were reported as the frequency (%) and mean$\pm$standard deviation (SD), respectively. The Chi-Square test was used for statistical analysis to assess the relationship between qualitative variables and One-Way analysis of variance (ANOVA) or sample t-test was used to examine the means of quantitative variables among separate groups. Anxiety ratings were classified based on severity, with individuals in the first group (no or minimum anxiety) serving as a control group. Also, a Cox regression model was developed for evaluation of the CAD and anxiety as an independent variable. Moreover, data was adjusted by age, sex, marital status, smoking status, job, and education levels. Every analysis was two-sided, and a p-value of 0.05 or lower was regarded as significant. Graph pad prism was used for drawing figures

## Results

Population characteristics are comprehensively demonstrated in Table 1. 60.4% of the sample were female, approximately 35.8% were considered moderate to highly educated, and 52.7% had low education levels. Most participants were non-smokers (70.6%) and married (94.1%). Regarding the results of the BAI, individuals were divided into 4 different groups at the time of admission into our study (Table 1) [16]. 58.4% of subjects were healthy for anxiety, whereas 5.6% were classified as having severe forms of anxiety (Table 1). Most healthy participants were employees (66%). However, the prevalence of moderate anxiety in unemployed subjects was significantly higher than other groups, and it was 19.2% (Table 1).

**Fig. 2:**
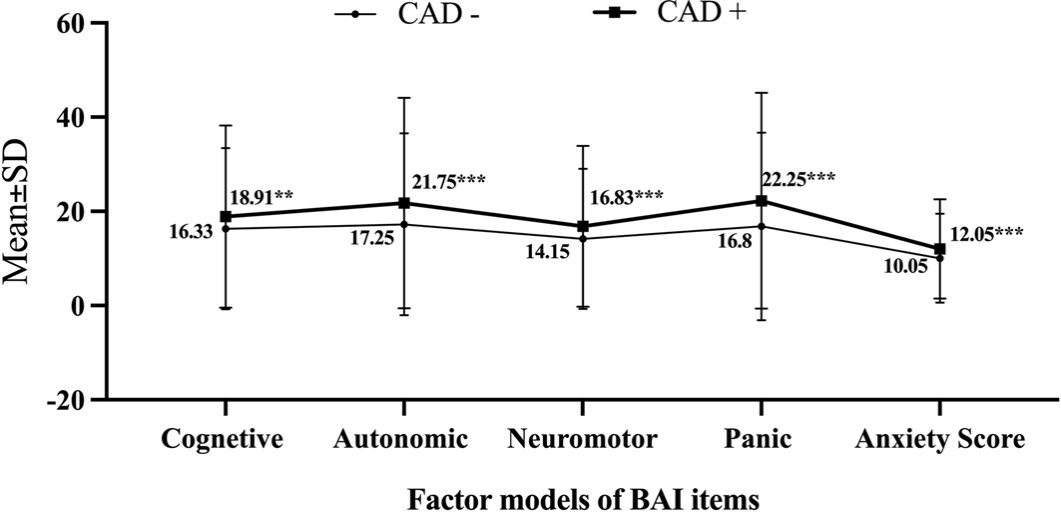
Mean of anxiety total score and factor models of BAI items in MASHAD cohort study according to the CAD event after 10 years follow-up; ***p < 0.001

The overall anxiety scores were evaluated at the baseline of the study among all participants including healthy individuals (10.05± 9.45) as well as participants who experienced coronary artery events during our 10-year follow-up (12.05±10.53) (Fig. 2). All individuals were classified as one property of the four anxiety structure model which includes cognitive, autonomic, neuromotor, and panic, by considering their answers to the 21 questions of BAI (Fig. 2) [20]. For simplicity and clearness, the results of each category were calculated as percentages and expressed as mean ± standard deviation (Fig. 2).

**Fig. 3:**
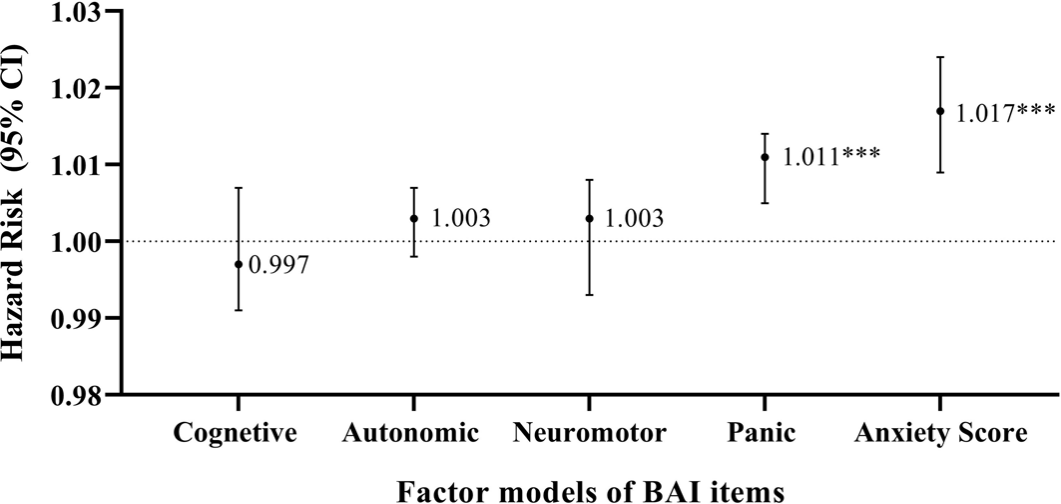
Relative risk of CAD event according to anxiety total score and factor models of BAI items in MASHAD study at baseline; sex, age, marriage status, job status, education level, smoking status, obesity, diabetes. ***p < 0.001

The hazard risk of developing CAD over a 10-year follow-up was calculated after adjustment for potential confounding variables including age, sex, marriage status, job, smoking status, education level, and having existing comorbidities such as diabetes, and obesity (Fig. 3). Our results showed a 1.7% risk of CAD (HR = 1.017, p-value < 0.001, 95% CI: 1.009–1.024) over 10 years with one unit increase in anxiety score (Fig. 3). Accordingly, based on the 4-factor model structure, we found that only panic disorder could significantly increase the risk (HR = 1.011, p-value < 0.001, 95%, CI: 1.005–1.014) of CAD by 1.1% over the 10-year follow-up (Fig. 3).

## Discussion

Regarding the possible deteriorating effects of anxiety in various cardiovascular diseases (CVDs) such as heart failure [21], CHD [22, 23], acute coronary syndrome (ACS) [24], and hypertension [25], investigating their interrelation is an important field of study. Various studies have indicated a prospective link between anxiety and CVD [26, 27], and one study has established a dose-response correlation between these two entities [28]. Accordingly, some studies have attributed the higher CAD incidence among patients with high anxiety levels to the accelerated hypothalamic-pituitary-adrenal axis, and the possible existence of a pro-atherosclerotic state in these patients [29, 30].

To the best of our knowledge, there is limited data about the association between the severity of anxiety symptoms and the risk of CAD incidence in initially healthy subjects among Iranians. Hosseini et al. utilized the BAI to evaluate anxiety scores in patients following new-onset myocardial infarction (MI) and found no relationship between anxiety and cardiac mortality during a 5-year follow-up [31]. In addition, Najafipour and colleagues established a meaningful correlation between anxiety and cardiac risk factors including opium usage and low physical activity [32]. Another investigation conducted by Abbasi et al. also determined an association between anxiety and various cardiac risk factors such as opium usage, positive family history of CAD, duration of CAD, hypertension, serum creatinine levels, and major adverse cardiac events (MACE) [33]. However, it is noteworthy that none of these studies have prospectively evaluated the effects of anxiety score severity on the incidennce rate of CAD, which directed us to investigate the possible correlation.

This study evaluated the correlation between anxiety and documented CAD of a well-characterized 10-year prospective cohort study among MASHAD cohort study. We found an increased based-line mean anxiety score among participants who experienced CAD during 10-year follow-up compared to the healthy group. This increase was seen in all properties of the four-factor model BAI which includes cognitive, autonomic, neuromotor, and panic. We observed a dose-response relationship between increased anxiety levels and CAD risk. Also, we showed that patients with higher anxiety levels were at a greater risk of CAD, in which every unit increase in the baseline BAI score was associated with a 1.7% higher risk of developing CAD over the next 10 years. In fact, in this study, we illustrated that anxiety was significantly associated with an increased risk of CAD, and we also found that anxiety total score and related disorders were meaningfully more severe among patients with established CAD.

The results of several meta-analyses confirmed that anxiety disorders increase the chance of subsequently developing CAD among healthy individuals [13, 27, 34]. Accordingly, our findings also showed a 1.7% increment in CAD incidence with every unit increase of anxiety score after adjustments for confounding variables such as age, sex, marriage status, job, smoking status, education level, and having existing comorbidities such as diabetes, and obesity. Additionally, a recent meta-analysis of 17 studies has evaluated the effects of anxiety on clinical outcomes among ACS patients. They reported that patients with concurrent ACS and anxiety are at a 47% greater risk of MACEs and a 21% higher risk of mortality. However, several studies did not find any association between anxiety disorders and clinical prognosis in CAD patients [35–38].

Various potential processes have been suggested to provide a reliable prediction for the correlation between anxiety disorders and CAD including cardiac arrhythmias [39], attenuated heart rate variability [40], and atherosclerosis development [30]. Although the exact mechanism by which anxiety may cause CAD is yet to be determined, several cardiac risk factors were found to be linked with augmented anxiety levels such as opium usage, positive family history of coronary artery diseases (CAD), duration of CAD, hypertension, low physical activity, serum creatinine levels, and major adverse cardiac events (MACE) [32, 33].

Multiple studies have shown a significant correlation between a variety of anxiety disorder categories and CAD [34, 41, 42], whereas some others have only reported meaningful correlations with panic disorder [13, 43, 44]. Similarly, we have found a significantly increased risk of developing CAD only within individuals experiencing panic disorder. In addition, as there is an overlap between symptoms of CAD and anxiety, and as CADs are thought to be more prevalent among patients diagnosed with anxiety [45], we also propose that underdiagnosis of cardiac diseases ought to be an area of concern in anxiety patients which might primarily occur due to falsely attributing cardiac symptoms to their underlying anxiety disorder.

**Fig. 4:**
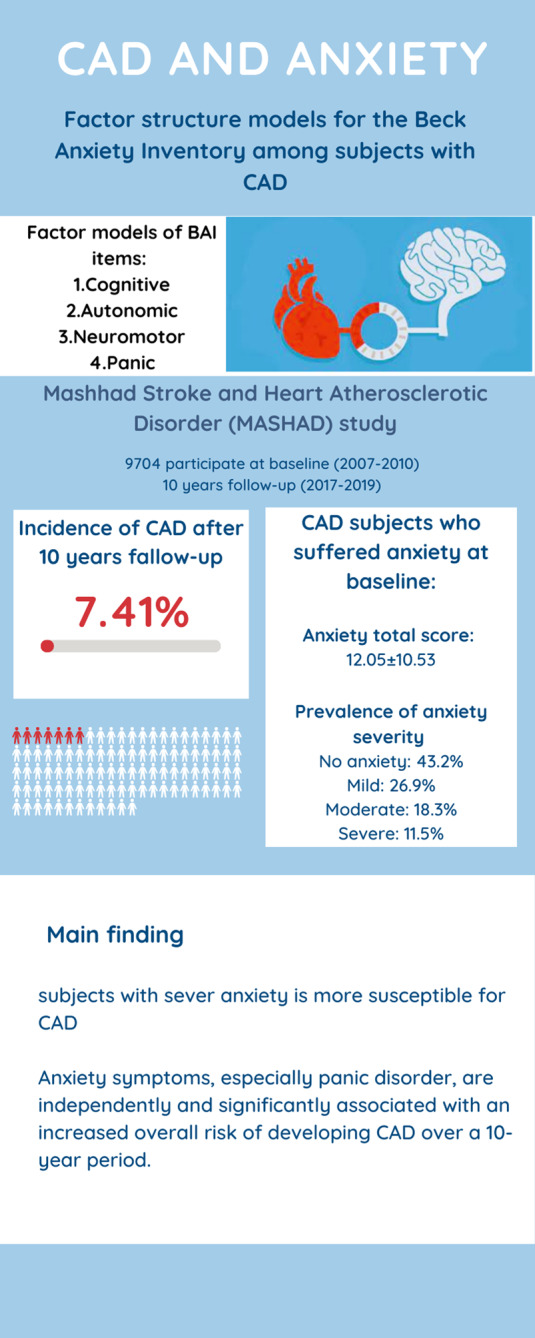
Summary of result

The strengths of this study were, Firstly, we utilized an immense sample of individuals as our baseline population which truly represented the general population of our target city. Secondly, we followed all participants for a long period of 10 years, and all of the cardiac events were documented and confirmed by expert academic cardiologists. Thirdly, our results are collected prospectively which makes it more competent to draw a conclusion.

The present study also comprises some limitations which we will discuss to guide future research. Firstly, even though we have excluded individuals with preexisting CAD at the baseline of our study, we are not able to exclude the odds of reverse causality due to a possible existing early-stage or asymptomatic CAD in our baseline sample. Secondly, our data showed that anxiety was correlated with several CAD risk factors such as obesity and diabetes. Although we have adjusted for these covariates in our analyses, there might be some other mediators missed in our considerations, such as having an unhealthy lifestyle, having simultaneous depression, or being a second-hand smoker.

## Conclusion

In conclusion, our 10-year prospective cohort study within the MASHAD cohort sheds light on the relationship between anxiety and the incidence of CAD (Fig. 4). We discovered that anxiety symptoms, especially panic disorder, are independently and significantly associated with an increased overall risk of developing CAD over a 10-year period. Based on our knowledge, recognition of subjects with panic disorder for a long time and treatment of them may decrease the risk of CAD. However, as there is a lack of evidence regarding the potential mechanisms, further studies are warranted to investigate the possible biological processes by which anxiety may cause CAD as well as proper interventions to slow down these mechanisms.

## Data Availability

The datasets used and/or analyzed during the current study are available from the corresponding author upon reasonable request.

## Abbreviations

ACS: Acute coronary syndrome
BAI: Beck Anxiety Inventory
BMI: Body mass index
CAD: Coronary artery disease
CHD: Coronary heart disease
CVD: Cardiovascular disease
DALY: Disability-adjusted life year
MACE: Major adverse cardiac event
MASHAD: Mashhad Stroke and Heart Atherosclerotic Disorder
MI: Myocardial infarction
SD: Standard deviation
